# Informing DSM-5: biological boundaries between bipolar I disorder, schizoaffective disorder, and schizophrenia

**DOI:** 10.1186/1741-7015-11-127

**Published:** 2013-05-14

**Authors:** Victoria E Cosgrove, Trisha Suppes

**Affiliations:** 1 Bipolar and Depression Research Program, VA Palo Alto Health Care System, 3801 Miranda Avenue (151T), Palo Alto, CA, 94304, USA; 2 Department of Psychiatry and Behavioral Sciences, Stanford University School of Medicine, Stanford, CA, USA

**Keywords:** Bipolar disorder, Hallucinations, Delusions, Schizoaffective disorder, Schizophrenia, DSM- 5, Genes, Psychiatric medication, Brain function

## Abstract

**Background:**

The fifth version of the Diagnostic and Statistical Manual of Mental Disorders (DSM-5) opted to retain existing diagnostic boundaries between bipolar I disorder, schizoaffective disorder, and schizophrenia. The debate preceding this decision focused on understanding the biologic basis of these major mental illnesses. Evidence from genetics, neuroscience, and pharmacotherapeutics informed the DSM-5 development process. The following discussion will emphasize some of the key factors at the forefront of the debate.

**Discussion:**

Family studies suggest a clear genetic link between bipolar I disorder, schizoaffective disorder, and schizophrenia. However, large-scale genome-wide association studies have not been successful in identifying susceptibility genes that make substantial etiological contributions. Boundaries between psychotic disorders are not further clarified by looking at brain morphology. The fact that symptoms of bipolar I disorder, but not schizophrenia, are often responsive to medications such as lithium and other anticonvulsants must be interpreted within a larger framework of biological research.

**Summary:**

For DSM-5, existing nosological boundaries between bipolar I disorder and schizophrenia were retained and schizoaffective disorder preserved as an independent diagnosis since the biological data are not yet compelling enough to justify a move to a more neurodevelopmentally continuous model of psychosis.

## Background

**Figure 1:**
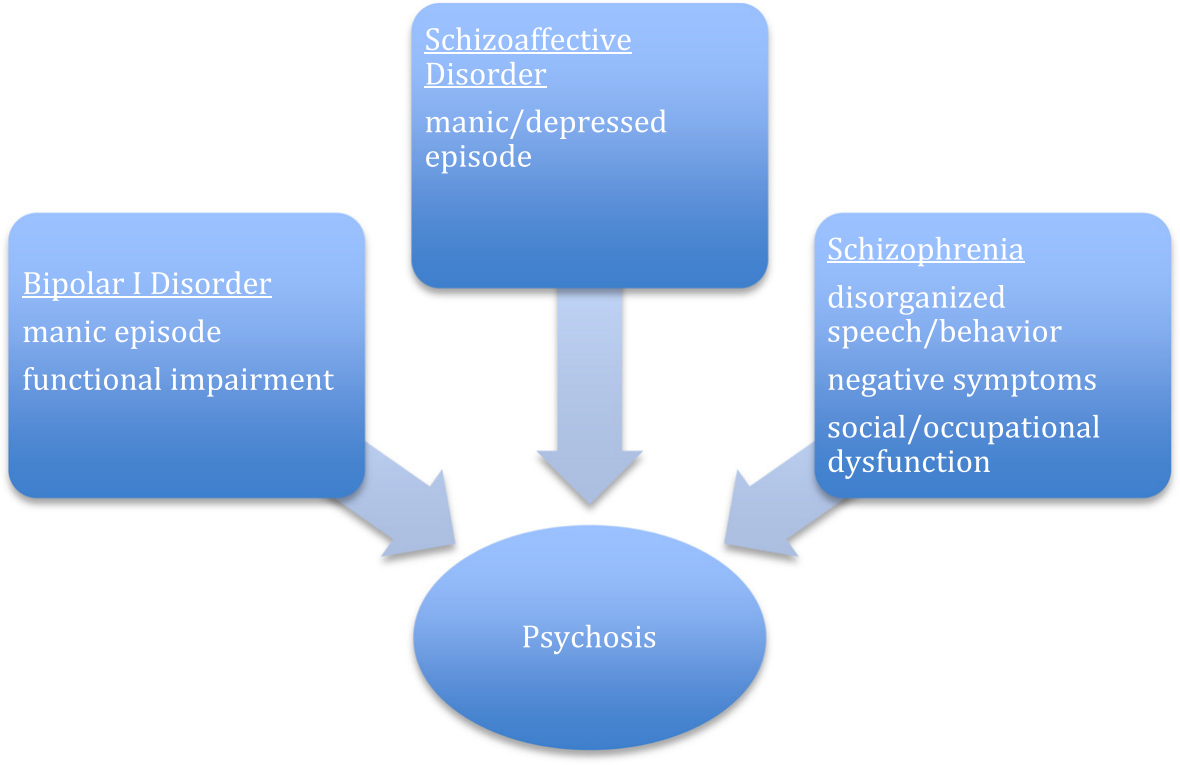
DSM-IV-TR features of bipolar I disorder, schizoaffective disorder, and schizophrenia.

Development of the fifth version of the Diagnostic and Statistical Manual of Mental Disorders (DSM-5), slated for publication in mid-2013, included a reconsideration of the relationship between psychosis occurring during major mental illness, specifically bipolar I disorder (BD I), schizoaffective disorder and schizophrenia. These discussions emerged before formal work on DSM-5 began based on critical review of the emerging data on the biological overlap between disorders seen particularly in genetics studies [1]. Historically, there has not been agreement about how biological research should best be interpreted to inform nosological boundaries specifically distinguishing psychotic disorders [2] and, more broadly, all psychiatric disorders [3-5]. On a phenotypic level, the lines of demarcation are concretely outlined in the current version of the DSM (DSM-IV-TR; see Figure 1), but the clinical features that distinguish disorders are often unclear or overlapping at the level of the presenting patient. Further, the DSM’s precise nosology [6] is often incompatible with first person experiences of mental illness [7].

Schizophrenia, which occurs in approximately 1% of the population, may be characterized by dramatic symptoms of delusions and hallucinations, affective flattening and amotivation, or negative symptoms. While individuals with schizophrenia may need ongoing support to maintain themselves independently, recovery initiatives have demonstrated that achievement of personal or professional goals and expansion of self-concept are attainable for individuals with schizophrenia [8,9]. By comparison, BD I occurs in about 1% of the population and is notable for its episodic nature with severe but periodic symptoms of mania and depression. A common manic presentation includes reports of minimal sleep accompanied by increased energy, changes in mood and judgment, and impulsivity. About 50% of manic episodes contain psychotic elements such as grandiosity, frank delusions and hallucinations, or paranoia [10]. Even in cases where manic episodes manifest psychotic content, many individuals may be responsive to medications and essentially return to full functioning with ongoing treatment. Schizoaffective disorder, estimated to occur in less than 1% of the population, appears to represent a midpoint on the pathologic spectrum between BD I and schizophrenia with psychotic symptoms predominant and mood symptoms of mania and depression less evident (see Figure 1) [11,12]. Individuals meeting criteria for this diagnosis report at least a two-week period without evidence of mood instability and persistent psychotic symptoms. In the DSM-IV TR categorization scheme, schizoaffective disorder includes both psychotic symptoms and severe mood episodes; however, by definition, there must be periods of psychosis without any disturbance in mood.

Hallucinations and delusions are typically considered the hallmark of schizophrenia and mood fluctuations central to BD I; however, psychotic symptoms may be present in both. Although bipolar mood episodes may have an inherent episodic rhythm, schizophrenia, schizoaffective disorder, and BD I can all be chronic, lifelong conditions that cause significant functional impairment.

Since both psychosis and mood disturbance may constitute core features of schizophrenia, BD I, and schizoaffective disorder, a debate arose during the early pre-DSM-5 development process about the idea of merging diagnoses in the revised manual [1]. A substantial body of research has focused on the genetic and neuroscientific etiological mechanisms of psychosis given that symptoms occur in schizophrenia in addition to schizoaffective disorder and BD I, among other psychiatric illnesses (major depression not being considered here) [13-15]. The argument in favor of merging diagnostic entities is based, in part, on the idea that schizoaffective disorder has proven to be a challenging differential diagnosis in clinical realms. Its diagnostic reliability across both clinicians and treatment settings is poor, and data promoting effective schizoaffective disorder-specific treatments are very limited [16].

Our aim in this paper is to first briefly and concisely review existing lines of biological evidence from behavioral and molecular genetics, neuroscience, and psychopharmacotherapeutics in order to determine whether they support or refute the idea of merging diagnoses involving psychosis in DSM-5. Given that DSM-5 has chosen to retain DSM-IV-TR’s operative criteria for BD I, schizoaffective disorder, and schizophrenia, the subsequent discussion will in part emphasize some of the key factors that may have informed the decision to sustain separation of nosologic and diagnostic criteria for BD I, schizoaffective disorder, and schizophrenia. Revisions in DSM-5 to all psychiatric diagnoses were made only after balancing tensions in creating a manual of psychiatric nosology that both adheres to the medical model of psychiatry [4] and is at once accurate, useful, and contemporary [17-20].

## Discussion

It is helpful to consider competing nosological models involving mood and psychotic disorders before attempting to critically evaluate biological evidence. Kraepelin’s dichotomous classification of psychosis into dementia praecox and manic-depressive insanity has informed earlier iterations of the DSM [21]. However, the National Institute of Mental Health’s (NIMH’s) Research Domain Criteria, or RDoC, may be a more useful lens through which to examine data linking biology and behavior in psychosis [22]. The RDoC framework purports a full spectrum, neurodevelopmentally continuous model for understanding psychiatric illness that is dimensional in nature and encourages assessment of behavior at genetic, molecular, cellular, and physiological levels. In other words, RDoC is a way to digest the relatively common findings that risk genes for one psychiatric disorder are associated with risk for many psychiatric disorders or that similar changes in brain structure or function are observed in many psychiatric disorders [23].

One way to conceptualize the debate about whether or not to merge schizophrenia, schizoaffective disorder, and BD I is to consider whether biological evidence for a dimensional model of psychosis consistent with RDoC is currently strong enough to warrant such a dramatic change to the DSM-IV-TR nosological system featuring discrete categorical classifications of normal and abnormal behavior. A third alternative for DSM-5 was potentially to bridge categorical and dimensional classification strategies by including additional intermediate ‘spectra’ diagnoses [24,25]. Biological evidence from the domains of behavioral and molecular genetics and brain morphology and functioning were considered. Additionally, psychopharmacotherapeutics, or differential response patterns to psychiatric medications for psychosis (that is, mood stabilizers, antipsychotics), were interpreted within the broader framework of biological mediators and moderators of treatment response (Tables 1 and 2).

**Table 1:** Summary of key evidence at the forefront of the boundaries of schizophrenia, schizoaffective disorder, and bipolar I disorder debate

**Evidence**	**Conclusion**
Family studies	
Tsuang *et al*., 1980 [26]; Mortensen *et al*., 2003 [27]; Lichtenstein *et al*., 2009 [28]; Van Snellenberg *et al*., 2009 [29]; Dean *et al*., 2010 [30]	Increased risk for bipolar disorder in families of individuals with schizophrenia and for schizophrenia in families of individuals with bipolar disorder
Gershon *et al*., 1988 [31]; Maier *et al*. 1993 [32]	Schizophrenia and bipolar disorder linked to unipolar depression
Kendler *et al*., 1998 [33]	Roscommon Family Study; vulnerability to psychosis may extend across schizophrenia, major depression, and bipolar disorder
Twin studies	
Cardno *et al*., 2002 [34]	Significant genetic correlations between schizophrenia, schizoaffective disorder, and mania
Genome wide association studies (GWAS)	
O’Donovan *et al*., 2009 [14]; Green *et al*., 2009 [35]; Williams *et al*., 2010 [36]; Lee *et al*., 2012 [37]	*ZNF804A* and *CACNA1C* may influence risk for both schizophrenia and bipolar disorder
Brain morphology	
Ellison-Wright and Bullmore, 2010 [38]; Arnone *et al*., 2009 [39]; Rimol *et al*., 2012 [40]	Some overlapping white and gray matter deficits, but cortical reductions exclusive to schizophrenia
McIntosh *et al*., 2006 [41]	Genetic liability for gray matter reductions in DLPRC and VLPFC exclusively in schizophrenia
Hulshoff *et al*., 2012 [42]	Overlapping white matter volume and areas of thin cortex suggest shared neurodevelopment
Pharmacotherapeutics	
Suppes *et al*., 1991 [43]; Schulz *et al*., 1999 [44]; Baldessarini *et al*., 1999 [45]; Leucht *et al*., 2006 [46]	Lithium can be used as monotherapy or for augmentation of antipsychotics in bipolar disorder but ineffective in schizophrenia
Casey *et al*., 2003 [47]	Divalproex prescribed for acute mania but minimal efficacy in schizophrenia
Tiihonen *et al*., 2003 [48]; Kremer *et al*., 2004 [49]; Zoccali *et al*., 2007 [50]; Goff *et al*., 2007 [51]	Initial reports of lamotrigine positive in add-on schizophrenia, but no better than placebo in multicenter, randomized trials
Post *et al*., 1999 [52]	Unexpected broad efficacy across psychotic disorders for second generation antipsychotics

**Table 2:** Strength of evidence for biological factors supporting merging in some way schizophrenia, schizoaffective and bipolar I disorder

**Biological factor**	**Strength of evidence**
Genetics—family studies	Strongest
Genetics—twin studies	Moderate
Genetics—candidate gene*/*GWAS	Moderate
Brain—morphology	Moderate
Pharmacotherapeutics—lithium, divalproex, lamotrigine, antipsychotics	Weak

GWAS, genome wide association studies.

### Genetic evidence

Genetic investigations offer a unique vantage point from which to consider the shared etiology of psychotic disorders. Aggregation within families of both schizophrenia and bipolar disorder has long been proposed as proof for continuity between the two disorders, and indeed its evidence spans multiple decades and is strong. Bipolar disorder, it seems, occurs more frequently than expected by chance in families of affected individuals and vice versa [26,29,30]. This same finding is observed in entire nations. Two large and important population-based studies—one based on data from the Danish Civil Registration System [27] and the other from the multi-generation and hospital-based registers in Sweden [28] both concluded that first-degree relatives of individuals with bipolar disorder were at higher risk for schizophrenia as well as bipolar disorder in several million families.

Twin studies provide further insight. Since monozygotic twins share 100% of their genes but dizygotic twins only 50%, on average, behavioral differences between the two can largely be attributed to environmental influences. The Maudsley Twin Registry studies are the only scientific investigation specifically focused on disentangling the genetic and environmental influences on different types of psychosis [34]. Findings confirm a shared genetic liability between psychosis in schizophrenia, schizoaffective disorder, and bipolar I mania. Additionally, genetic contributions to schizoaffective disorder appear to be entirely shared with those contributing to schizophrenia and mania, shedding substantial doubt on the accuracy of an independent schizoaffective disorder diagnosis [53].

Given the robust evidence of shared genetic etiology between schizophrenia and bipolar disorder amassed from family studies, a ‘hopeful’ energy drove the search for specific candidate genes related to psychosis in the late 1990s and early 2000s. However, this exploration—first using single-gene association methodology and later, genome-wide association studies (GWAS)—has proven difficult and largely yielded disappointing and inconclusive findings [54]. It has not been challenging to identify genetic variants common to both schizophrenia-spectrum and bipolar disorders; however, their relative etiological contributions seem to be very small. In recent years, two risk genes have repeatedly emerged as critical and common to psychosis in both disorders. First, an intron of zinc finger binding protein 804A (*ZNF804A*) on chromosome 6, a protein sequence potentially involved in brain connectivity, has been implicated. Based on odds ratios, *ZNF804A* appears to act as a susceptibility site for psychosis although its contribution is likely very small [14,37]. Second, an intron of the L-type voltage dependent calcium channel alpha 1C subunit (*CACNA1 C*), potentially involved in neuronal calcium-dependent processes, has also repeatedly been identified as a gene conferring a small but detectable increased risk in both schizophrenia and bipolar disorder [55]

One glaring criticism of many genetic investigations has been that very few make a phenotypic distinction between psychotic and non-psychotic BD I when making comparisons with schizophrenia. For example, Green and colleagues [55] report that 66% of their bipolar disorder cases endorsed a positive history of psychotic symptoms; however, their subsequent genetic analyses involving *CACNA1 C* do not differentiate this subgroup. Since psychotic symptoms occur generally in about 50% of manic episodes of BDI, it is difficult to know whether a susceptibility locus such as *CACNA1 C* confers risk for psychosis or other features shared between the two disorders (that is., anhedonia, cognitive impairment, and so on).

### Brain morphology

Evidence from investigations of brain morphology does little to clarify the boundaries between various psychotic disorders. Rather, it seems that in addition to some disorder-specific changes, psychosis occurring as a result of BD I or schizophrenia appears to be related to patterns of morphological changes in brain regions that seem to be involved in both of these disorders [15]. While reductions in cortical volume and thickness appear to be specific for schizophrenia, and not BD I [40], decreases in total brain mass have been reported in both disorders [39]. Further, consonant gray matter reductions in paralimbic regions including the anterior cingulate and insula, thought to be involved in emotional processing, have been observed in schizophrenia and bipolar disorder [56]. Again, none of these studies differentiates between psychotic and non-psychotic BD I, and some even fail to differentiate between bipolar I and bipolar II, a form of the illness not involving manic episodes and with less psychotic burden of these disorders.

Combining family-based behavioral genetic methodologies with brain morphometry techniques has led to findings that in part point to shared biological origins, although there remains confusion. While two recent studies suggest that prefrontal cortical grey matter reductions [41] and reduced hippocampal volumes [57] may be correlated to increased genetic susceptibility to schizophrenia but not BD I, others suggest shared genetic liabilities for potentially pathognomic factors that may affect differing brain regions and networks. McDonald and colleagues observed that both schizophrenia and bipolar disorder were related to white matter deficits in overlapping regions of the brain but that deficits in grey matter appeared in completely separate regions [58]. It is worth noting that their sample of individuals with bipolar disorder consisted only of those who had experienced psychotic symptoms. By far, the most convincing evidence linking genetic susceptibility and brain structure was reported in a recent twin study of monozygotic and dizygotic twin pairs concordant or discordant for schizophrenia or bipolar disorder [42]. Absence of psychosis was not exclusionary, but genetic liability for both disorders was associated with reduction in white matter volume as well as thinner areas of the cortex in similar areas of the brain.

### Pharmacotherapeutics

Response patterns to medication across different psychiatric diagnostic categories are complex. There is more than one clear case, for example, of medications being fully effective to treat all symptoms including mania and psychosis for BD I and ineffective to treat patients with schizophrenia or schizoaffective disorder. As well, individuals exhibiting the same diagnostic profile and with similar presenting symptoms may respond differently to the same medications. There are still too few clear guideposts to predict optimal treatment response. Psychophamacological response data interpreted in isolation are inherently inferential in nature and thus, must be comprehended with caution. Interpretation must be integrated within a larger framework of research that defines underlying mediators or moderators of treatment response, such as behavioral or molecular genetic profiles, neuroanatomy or brain functioning. Importantly, in this section we have elected to discuss clinically observed and studied impacts of medications in broad use that highlight differences across current diagnostic categories. We will not discuss cellular receptor differences between these different medications as these are beyond the scope of this manuscript. For review and discussion of purported medication mechanisms, we refer you to Steven Stahl’s *Essential Psychopharmacology* work [59].

One such example of different response patterns across psychotic disorders is lithium, approved by the Food and Drug Administration (FDA) in 1971 for treatment of mania and soon afterward considered a first-line treatment for bipolar disorder [60]. Despite clear strong effectiveness studies in BD I, lithium utilized as monotherapy or as augmentation of antipsychotic medication for individuals with schizophrenia appears to be largely ineffective [61,62]. A pivotal study analyzing recurrence of bipolar episodes following discontinuation of lithium maintenance treatment demonstrated that patients relapsed into mania or depression more quickly following lithium discontinuation than the individual’s normal course of illness might predict [43]. In other words, patients with bipolar disorder tend to show ‘rebound’ effects from abrupt discontinuation of lithium whereas patients with schizophrenia treated with lithium do not [45].

Divalproex, an anticonvulsant, was introduced by the FDA in 1995 for treatment of BD I mania. Similar to lithium, divalproex has minimal benefit in the treatment of schizophrenia or schizoaffective disorder. In combination with olanzapine and risperidone, divalproex resulted in an accelerated, initial decrease in psychotic symptoms [47]. However, a recent Cochrane analysis concluded that there were no available data to substantiate the use of divalproex as monotherapy in schizophrenia [61].

Response to lamotrigine in different psychotic disorders is also pertinent to a discussion about potentially merging schizophrenia and BD I. Lamotrigine was approved by the FDA in 2003 for the prevention of new episodes of mania or depression in BD I [62]. Although early reports of adjunctive use of lamotrigine to treat schizophrenia were positive [48], it was, in fact, shown to be no more efficacious than placebo (as an add-on agent) in two recently conducted trials [51].

Finally, the use of both typical and second generation (atypical) antipsychotics in the treatment of various psychotic disorders should be considered. From the 1960s to 1980s, before lithium was approved by the FDA and widely used, typical antipsychotics, such as haloperidol or fluphenazine, were generally regarded as the only available first-line medications for the treatment of mania [63]. Some evidence suggests that patients with BD I treated with typical antipsychotics may be more sensitive to serious side effect profiles including neuroleptic malignant syndrome than patients with schizophrenia [52]. Because of unexpected, broad effectiveness and—at least before potential metabolic side effects were noted—comparably favorable side effect profiles, second generation antipsychotics are frequent choices in schizophrenia, schizoaffective disorder, and BD I. At a minimum, all work reasonably well as antipsychotic agents in treating these disorders, despite acting across a range of receptor systems (for example, serotonin, dopaminergic, and so on), and having heterogenous side effect profiles.

## Summary

With regard to DSM-5, the biological data are not yet compelling enough to warrant embracing a more neurodevelopmentally continuous model of psychosis consistent with RDoC and not yet strong enough on their own to currently warrant a radical change to psychiatric nosology, such as merging schizophrenia and psychotic BD I. For DSM-5, existing nosological boundaries between the two were retained and schizoaffective disorder preserved as an independent diagnosis. While a shared genetic liability among psychotic disorders is likely, the real biological evidence still largely stems from family studies and is not routinely supported by candidate gene or GWAS investigations. It is still not possible to make a definitive statement about what genes are primarily responsible for this genetic risk, since confirming roles for putative genes has not panned out on a molecular level in the way behavioral geneticists had hoped. GWAS findings have demonstrated likely small roles for *ZNF804A* and *CACNA1 C*; however, mechanistically these are not well understood.

Response to medication, an area of extensive research, indicates we do not yet understand the biological basis of these illnesses. Some researchers consider psychotic phenomena to be epiphenomena to the primary illness. Thus, under this idea, lithium treats the underlying condition in BD I, resolving psychotic manic symptoms, but is ineffective in schizophrenia given its inability to treat the underlying pathophysiology of this illness.

Even after linking genetic risk to both disorders with structural changes in the brain and considering response to psychotropic medications, the biological evidence falls short of the requisite durability necessary to warrant a DSM-5 change that will likely command diagnosis in both clinical practice and research investigations for at least a decade to come.

Still, in spite of the shortcomings of the existing biological evidence, an RDoC-inspired model for psychosis integrating evidence from multiple modalities seems probable for DSM revisions of the future. To what extent these lines of evidence will influence future psychiatric nosology depends largely on how our understanding of brain function changes as science advances. As technology develops, it is to be hoped it will become easier and cheaper to investigate the complex alliances between brain circuits and genes that lead to the neurodevelopment of psychosis. Clear, replicable phenotyping of illness characteristics will be most critical to these efforts.

## Abbreviations

BD I: bipolar I disorder; DSM-5: Diagnostic and Statistical Manual of Mental Disorders, 5th edition; FDA: Food and Drug Administration; GWAS: genome wide association studies; RDoC: research domain criteria.

## Competing interests

In the calendar years from 2008–2013, Trisha Suppes has: received research funding or medication support from Abbott Laboratories, AstraZeneca, JDS Pharmaceuticals, NIMH, Pfizer, the Stanley Medical Research Institute, Pfizer Inc., Sunovion Pharmaceuticals Inc., Elan Pharma International Limited, VA Cooperative Studies Program; served in a consulting or advisory capacity to Orexigen Therapeutics and Sunovion Pharmaceuticals Inc; has received royalties from Jones and Bartlett; served on the speakers bureau for AstraZeneca; received honoraria from CME Outfitters, Medscape, Wolter Kluwer Pharma Solutions, and Continuing Medical Education, HealthmattersCME; and received travel funds from AstraZeneca and Sunovion Pharmaceuticals Inc. VC declares no competing interests.

## Authors’ contributions

VC participated in the Debate’s design and drafted the manuscript. TS initiated the Debate’s design, assisted in drafting the manuscript, and provided extensive critical review. VC and TS read and approved the final manuscript.

## Pre-publication history

The pre-publication history for this paper can be accessed here:

http://www.biomedcentral.com/1741-7015/11/127/prepub
